# (*E*)-*N*′-(4-Meth­oxy­benzyl­idene)-3-nitro­benzohydrazide

**DOI:** 10.1107/S1600536811025554

**Published:** 2011-07-02

**Authors:** Jin-Wu Guo, Jun-Ying Ma, Chao-Wei Sun

**Affiliations:** a Chemical Engineering & Pharmaceutics College, Henan University of Science and Technology, Luoyang Henan 471003, People’s Republic of China

## Abstract

In the title compound, C_15_H_13_N_3_O_4_, the two substituted benzene rings form a dihedral angle of 5.0 (3)°. In the crystal, inter­molecular N—H⋯O hydrogen bonds link mol­ecules into chains along the *b* axis.

## Related literature

For background to the binding properties and biological activity of condensation products of aldehydes with benzohydrazides, see: Sanchez-Lozano *et al.* (2011 ▶); Wang (2011 ▶); Cui *et al.* (2011 ▶); Zhu (2011 ▶); Peng (2011 ▶). For related structures, see: Hashemian *et al.* (2011 ▶); Shalash *et al.* (2010 ▶).
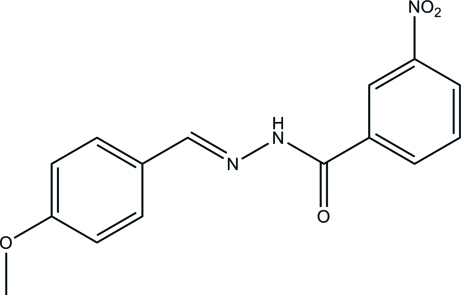


## Experimental

### 

#### Crystal data

C_15_H_13_N_3_O_4_
*M* *_r_* = 299.28
Monoclinic, 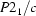

*a* = 13.667 (4) Å
*b* = 4.889 (3) Å
*c* = 22.522 (4) Å
β = 104.113 (3)°
*V* = 1459.5 (10) Å^3^
*Z* = 4
Mo *K*α radiation
μ = 0.10 mm^−1^
*T* = 298 K
0.30 × 0.28 × 0.27 mm

#### Data collection

Bruker SMART APEX CCD area-detector diffractometer
Absorption correction: multi-scan (*SADABS*; Bruker, 2007 ▶) *T*_min_ = 0.970, *T*_max_ = 0.973
9043 measured reflections
2981 independent reflections
1493 reflections with *I* > 2σ(*I*)
*R*_int_ = 0.073

#### Refinement

*R*[*F* ^2^ > 2σ(*F* ^2^)] = 0.063
*wR*(*F* ^2^) = 0.173
*S* = 1.02
2981 reflections
200 parameters
H-atom parameters constrained
Δρ_max_ = 0.19 e Å^−3^
Δρ_min_ = −0.26 e Å^−3^

### 

Data collection: *SMART* (Bruker, 2007 ▶); cell refinement: *SAINT* (Bruker, 2007 ▶); data reduction: *SAINT*; program(s) used to solve structure: *SHELXS97* (Sheldrick, 2008 ▶); program(s) used to refine structure: *SHELXL97* (Sheldrick, 2008 ▶); molecular graphics: *SHELXTL* (Sheldrick, 2008 ▶); software used to prepare material for publication: *SHELXTL*.

## Supplementary Material

Crystal structure: contains datablock(s) global, I. DOI: 10.1107/S1600536811025554/rz2619sup1.cif

Structure factors: contains datablock(s) I. DOI: 10.1107/S1600536811025554/rz2619Isup2.hkl

Supplementary material file. DOI: 10.1107/S1600536811025554/rz2619Isup3.cml

Additional supplementary materials:  crystallographic information; 3D view; checkCIF report

## Figures and Tables

**Table 1:** Hydrogen-bond geometry (Å, °)

*D*—H⋯*A*	*D*—H	H⋯*A*	*D*⋯*A*	*D*—H⋯*A*
N2—H2*A*⋯O2^i^	0.86	2.13	2.895 (3)	148

Symmetry code: (i) 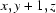.

**Fig. 1.**
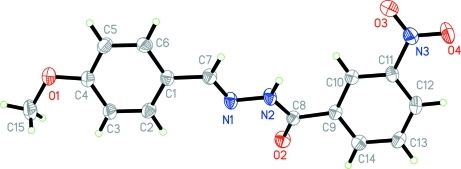
The molecular structure of the title compound with displacement ellipsoids shown at 30% probability level.

**Fig. 2.**
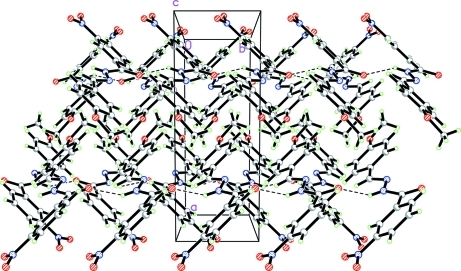
Packing diagram of the title compound, viewed along the c axis. Hydrogen bonds are indicated by dashed lines.

## supplementary crystallographic information

### Comment

The compounds derived from the condensation reaction of aldehydes with benzohydrazides play a vital role in coordination chemistry due to their metal binding property (Sanchez-Lozano *et al.*, 2011; Wang, 2011; Cui *et al.*, 2011). Moreover, most of such compounds possess effective biological activity (Zhu, 2011; Peng, 2011). In recent years, a number of such compounds have been reported (Hashemian *et al.*, 2011; Shalash *et al.*, 2010). In this paper, the title new compound, (*E*)-*N*'-(4-Methoxybenzylidene)-3-nitrobenzohydrazide, (I), is reported.

The molecular structure of (I) is shown in Fig. 1. The bond lengths in (I) are normal and comparable with those observed in the reported structures cited above. The two substituted benzene rings form a dihedral angle of 5.0 (3)°. In the crystal, intermolecular N–H···O hydrogen bonds link molecules into one-dimensional chains along the *b* axis (Fig. 2; Table 1).

### Experimental

4-Methoxybenzaldehyde (0.136 g, 1 mmol), 3-nitrobenzohydrazide (0.181 g, 1 mmol), and a few drops of acetic acid were mixed in methanol (30 ml). The solution was magnetically stirred at ambient temperature for 10 min until it turned to yellow. The solution was slowly evaporated in open air to give needle-shaped pale yellow single crystals.

### Refinement

H atoms were placed in idealized positions (C–H = 0.93-0.96 Å, N–H = 0.86 Å), and refined as riding, with *U*_iso_(H) = 1.2*U*_eq_(C, N) or 1.5*U*_eq_(C) for methyl H atoms.

### Crystal data

**Table:**

C_15_H_13_N_3_O_4_	*F*(000) = 624
*M**_r_* = 299.28	*D*_x_ = 1.362 Mg m^−^^3^
Monoclinic, *P*2_1_/*c*	Mo *K*α radiation, λ = 0.71073 Å
Hall symbol: -P 2ybc	Cell parameters from 762 reflections
*a* = 13.667 (4) Å	θ = 2.6–24.3°
*b* = 4.889 (3) Å	µ = 0.10 mm^−^^1^
*c* = 22.522 (4) Å	*T* = 298 K
β = 104.113 (3)°	Needle fragment, pale yellow
*V* = 1459.5 (10) Å^3^	0.30 × 0.28 × 0.27 mm
*Z* = 4	

### Data collection

**Table:**

Bruker SMART APEX CCD area-detector diffractometer	2981 independent reflections
Radiation source: fine-focus sealed tube	1493 reflections with *I* > 2σ(*I*)
graphite	*R*_int_ = 0.073
ω scans	θ_max_ = 26.5°, θ_min_ = 2.1°
Absorption correction: multi-scan (*SADABS*; Bruker, 2007)	*h* = −16→16
*T*_min_ = 0.970, *T*_max_ = 0.973	*k* = −6→6
9043 measured reflections	*l* = −28→28

### Refinement

**Table:**

Refinement on *F*^2^	Primary atom site location: structure-invariant direct methods
Least-squares matrix: full	Secondary atom site location: difference Fourier map
*R*[*F*^2^ > 2σ(*F*^2^)] = 0.063	Hydrogen site location: inferred from neighbouring sites
*wR*(*F*^2^) = 0.173	H-atom parameters constrained
*S* = 1.02	*w* = 1/[σ^2^(*F*_o_^2^)] where *P* = (*F*_o_^2^ + 2*F*_c_^2^)/3
2981 reflections	(Δ/σ)_max_ < 0.001
200 parameters	Δρ_max_ = 0.19 e Å^−^^3^
0 restraints	Δρ_min_ = −0.26 e Å^−^^3^

### Special details

**Table:**

Geometry. All e.s.d.'s (except the e.s.d. in the dihedral angle between two l.s. planes) are estimated using the full covariance matrix. The cell e.s.d.'s are taken into account individually in the estimation of e.s.d.'s in distances, angles and torsion angles; correlations between e.s.d.'s in cell parameters are only used when they are defined by crystal symmetry. An approximate (isotropic) treatment of cell e.s.d.'s is used for estimating e.s.d.'s involving l.s. planes.
Refinement. Refinement of *F*^2^ against ALL reflections. The weighted *R*-factor *wR* and goodness of fit *S* are based on *F*^2^, conventional *R*-factors *R* are based on *F*, with *F* set to zero for negative *F*^2^. The threshold expression of *F*^2^ > σ(*F*^2^) is used only for calculating *R*-factors(gt) *etc*. and is not relevant to the choice of reflections for refinement. *R*-factors based on *F*^2^ are statistically about twice as large as those based on *F*, and *R*- factors based on ALL data will be even larger.

### Fractional atomic coordinates and isotropic or equivalent isotropic displacement parameters (Å^2^)

**Table:**

	*x*	*y*	*z*	*U*_iso_*/*U*_eq_	
N1	0.26856 (19)	0.4077 (5)	0.12291 (10)	0.0478 (7)	
N2	0.22236 (19)	0.4793 (4)	0.06262 (10)	0.0481 (7)	
H2A	0.2022	0.6443	0.0537	0.058*	
N3	−0.03766 (19)	0.8680 (5)	−0.13175 (12)	0.0519 (7)	
O1	0.44934 (18)	0.4662 (5)	0.41484 (9)	0.0673 (7)	
O2	0.23532 (17)	0.0453 (4)	0.02765 (9)	0.0572 (6)	
O3	−0.05412 (17)	0.9975 (4)	−0.08818 (10)	0.0658 (7)	
O4	−0.08122 (19)	0.9108 (5)	−0.18561 (10)	0.0813 (8)	
C1	0.3188 (2)	0.5554 (6)	0.22758 (11)	0.0425 (7)	
C2	0.3912 (2)	0.3558 (6)	0.24755 (12)	0.0486 (8)	
H2	0.4092	0.2423	0.2188	0.058*	
C3	0.4377 (2)	0.3199 (6)	0.30896 (12)	0.0501 (8)	
H3A	0.4870	0.1862	0.3210	0.060*	
C4	0.4100 (2)	0.4862 (6)	0.35258 (12)	0.0467 (8)	
C5	0.3381 (3)	0.6875 (6)	0.33375 (13)	0.0543 (9)	
H5	0.3197	0.7990	0.3627	0.065*	
C6	0.2933 (2)	0.7246 (6)	0.27212 (12)	0.0510 (8)	
H6	0.2459	0.8628	0.2600	0.061*	
C7	0.2695 (2)	0.5983 (6)	0.16229 (12)	0.0470 (8)	
H7	0.2388	0.7651	0.1495	0.056*	
C8	0.2095 (2)	0.2863 (6)	0.01832 (12)	0.0417 (7)	
C9	0.1590 (2)	0.3825 (5)	−0.04553 (11)	0.0399 (7)	
C10	0.0857 (2)	0.5890 (5)	−0.05782 (12)	0.0410 (7)	
H10	0.0681	0.6832	−0.0261	0.049*	
C11	0.0400 (2)	0.6497 (6)	−0.11841 (12)	0.0421 (7)	
C12	0.0651 (3)	0.5157 (6)	−0.16723 (13)	0.0523 (8)	
H12	0.0331	0.5608	−0.2074	0.063*	
C13	0.1384 (2)	0.3145 (6)	−0.15503 (12)	0.0541 (9)	
H13	0.1569	0.2247	−0.1870	0.065*	
C14	0.1841 (2)	0.2474 (6)	−0.09470 (12)	0.0492 (8)	
H14	0.2324	0.1096	−0.0868	0.059*	
C15	0.5296 (3)	0.2752 (9)	0.43634 (14)	0.0875 (12)	
H15A	0.5063	0.0935	0.4243	0.131*	
H15B	0.5512	0.2849	0.4802	0.131*	
H15C	0.5852	0.3190	0.4189	0.131*	

### Atomic displacement parameters (Å^2^)

**Table:**

	*U*^11^	*U*^22^	*U*^33^	*U*^12^	*U*^13^	*U*^23^
N1	0.0634 (18)	0.0364 (15)	0.0395 (13)	0.0020 (13)	0.0045 (12)	0.0084 (11)
N2	0.0673 (19)	0.0320 (14)	0.0397 (14)	0.0072 (13)	0.0030 (12)	0.0094 (10)
N3	0.0450 (17)	0.0560 (18)	0.0523 (17)	−0.0011 (14)	0.0073 (13)	0.0085 (13)
O1	0.0695 (17)	0.0890 (18)	0.0390 (12)	0.0124 (14)	0.0046 (11)	−0.0008 (11)
O2	0.0809 (17)	0.0307 (12)	0.0582 (13)	0.0075 (11)	0.0133 (12)	0.0063 (9)
O3	0.0615 (16)	0.0655 (16)	0.0698 (15)	0.0145 (12)	0.0146 (13)	0.0037 (12)
O4	0.0762 (19)	0.105 (2)	0.0522 (14)	0.0205 (15)	−0.0048 (13)	0.0249 (13)
C1	0.050 (2)	0.0380 (18)	0.0387 (16)	−0.0020 (15)	0.0093 (14)	0.0047 (12)
C2	0.062 (2)	0.0399 (18)	0.0441 (17)	0.0041 (16)	0.0133 (15)	−0.0018 (13)
C3	0.055 (2)	0.052 (2)	0.0405 (16)	0.0077 (16)	0.0058 (14)	0.0046 (14)
C4	0.047 (2)	0.051 (2)	0.0394 (17)	−0.0044 (16)	0.0072 (14)	0.0016 (13)
C5	0.068 (2)	0.054 (2)	0.0424 (17)	0.0057 (18)	0.0169 (16)	−0.0048 (14)
C6	0.054 (2)	0.0454 (19)	0.0536 (18)	0.0073 (16)	0.0128 (15)	0.0032 (14)
C7	0.059 (2)	0.0365 (18)	0.0443 (17)	0.0038 (16)	0.0107 (15)	0.0060 (13)
C8	0.0498 (19)	0.0344 (18)	0.0416 (15)	0.0004 (15)	0.0123 (14)	0.0039 (13)
C9	0.0529 (19)	0.0271 (15)	0.0400 (15)	−0.0051 (14)	0.0118 (14)	0.0011 (12)
C10	0.0461 (19)	0.0353 (17)	0.0417 (16)	−0.0040 (14)	0.0109 (14)	0.0007 (12)
C11	0.0452 (19)	0.0382 (17)	0.0411 (16)	−0.0049 (15)	0.0070 (14)	0.0064 (12)
C12	0.060 (2)	0.057 (2)	0.0356 (16)	−0.0111 (18)	0.0034 (15)	0.0027 (14)
C13	0.070 (2)	0.053 (2)	0.0422 (17)	−0.0027 (18)	0.0203 (16)	−0.0022 (15)
C14	0.057 (2)	0.0402 (19)	0.0511 (18)	−0.0007 (16)	0.0152 (15)	0.0005 (14)
C15	0.093 (3)	0.105 (3)	0.049 (2)	0.024 (3)	−0.013 (2)	0.001 (2)

### Geometric parameters (Å, °)

**Table:**

N1—C7	1.284 (3)	C5—C6	1.386 (4)
N1—N2	1.395 (3)	C5—H5	0.9300
N2—C8	1.353 (3)	C6—H6	0.9300
N2—H2A	0.8600	C7—H7	0.9300
N3—O4	1.232 (3)	C8—C9	1.510 (4)
N3—O3	1.234 (3)	C9—C14	1.402 (3)
N3—C11	1.483 (4)	C9—C10	1.402 (4)
O1—C4	1.377 (3)	C10—C11	1.387 (3)
O1—C15	1.432 (4)	C10—H10	0.9300
O2—C8	1.233 (3)	C11—C12	1.393 (4)
C1—C2	1.385 (4)	C12—C13	1.383 (4)
C1—C6	1.408 (4)	C12—H12	0.9300
C1—C7	1.475 (4)	C13—C14	1.389 (4)
C2—C3	1.385 (4)	C13—H13	0.9300
C2—H2	0.9300	C14—H14	0.9300
C3—C4	1.397 (4)	C15—H15A	0.9600
C3—H3A	0.9300	C15—H15B	0.9600
C4—C5	1.382 (4)	C15—H15C	0.9600
			
C7—N1—N2	114.6 (2)	C1—C7—H7	119.7
C8—N2—N1	119.3 (2)	O2—C8—N2	124.1 (2)
C8—N2—H2A	120.4	O2—C8—C9	120.3 (2)
N1—N2—H2A	120.4	N2—C8—C9	115.6 (2)
O4—N3—O3	123.9 (3)	C14—C9—C10	118.9 (2)
O4—N3—C11	118.1 (3)	C14—C9—C8	117.6 (3)
O3—N3—C11	117.9 (2)	C10—C9—C8	123.4 (2)
C4—O1—C15	117.8 (2)	C11—C10—C9	118.5 (3)
C2—C1—C6	117.6 (2)	C11—C10—H10	120.8
C2—C1—C7	122.7 (3)	C9—C10—H10	120.8
C6—C1—C7	119.6 (3)	C10—C11—C12	122.5 (3)
C1—C2—C3	122.1 (3)	C10—C11—N3	118.8 (3)
C1—C2—H2	119.0	C12—C11—N3	118.7 (3)
C3—C2—H2	119.0	C13—C12—C11	118.9 (3)
C2—C3—C4	119.4 (3)	C13—C12—H12	120.5
C2—C3—H3A	120.3	C11—C12—H12	120.5
C4—C3—H3A	120.3	C12—C13—C14	119.6 (3)
O1—C4—C5	115.8 (3)	C12—C13—H13	120.2
O1—C4—C3	124.7 (3)	C14—C13—H13	120.2
C5—C4—C3	119.5 (3)	C13—C14—C9	121.5 (3)
C4—C5—C6	120.5 (3)	C13—C14—H14	119.2
C4—C5—H5	119.7	C9—C14—H14	119.2
C6—C5—H5	119.7	O1—C15—H15A	109.5
C5—C6—C1	120.7 (3)	O1—C15—H15B	109.5
C5—C6—H6	119.6	H15A—C15—H15B	109.5
C1—C6—H6	119.6	O1—C15—H15C	109.5
N1—C7—C1	120.7 (3)	H15A—C15—H15C	109.5
N1—C7—H7	119.7	H15B—C15—H15C	109.5

### Hydrogen-bond geometry (Å, °)

**Table:**

*D*—H···*A*	*D*—H	H···*A*	*D*···*A*	*D*—H···*A*
N2—H2A···O2^i^	0.86	2.13	2.895 (3)	148

Symmetry codes: (i) *x*, *y*+1, *z*.

## References

[bb1] Bruker (2007). *SMART*, *SAINT* and *SADABS* Bruker AXS Inc., Madison, Wisconsin, USA.

[bb2] Cui, Y.-M., Cai, Y.-J. & Chen, W. (2011). *J. Coord. Chem.* **64**, 1385–1392.

[bb3] Hashemian, S., Ghaeinee, V. & Notash, B. (2011). *Acta Cryst.* E**67**, o171. 10.1107/S1600536810052128 PMC3050232 21522678

[bb4] Peng, S.-J. (2011). *J. Chem. Crystallogr.* **41**, 280–285.

[bb5] Sanchez-Lozano, M., Vazquez-Lopez, E. M., Hermida-Ramon, J. M. & Estevez, C. M. (2011). *Polyhedron*, **30**, 953–962.

[bb6] Shalash, M., Salhin, A., Adnan, R., Yeap, C. S. & Fun, H.-K. (2010). *Acta Cryst.* E**66**, o3126–o3127. 10.1107/S1600536810045162 PMC3011781 21589430

[bb7] Sheldrick, G. M. (2008). *Acta Cryst.* A**64**, 112–122. 10.1107/S0108767307043930 18156677

[bb8] Wang, N. (2011). *Synth. React. Inorg. Met. Org. Nano-Met. Chem.* **41**, 378–383.

[bb9] Zhu, H.-Y. (2011). *Chin. J. Struct. Chem.* **30**, 724–730.

